# Investigating a Newly Developed Educational Orthopedic Application for Medical Interns in a Before-after Quasi-clinical Trial Study

**DOI:** 10.1186/s12909-021-02918-y

**Published:** 2021-09-29

**Authors:** Mahla Daliri B.O., Hassan M. Majd, Ali Moradi

**Affiliations:** 1 Orthopedic Research Center, Ghaem hospital, Mashhad University of Medical Sciences, Mashhad, Iran; 2 Research Development Center, Ghaem hospital, Mashhad University of Medical Sciences, Mashhad, Iran

**Keywords:** Medical education, education method, E-learning, Medical application, educational application, orthopedic application, smartphone medical learning

## Abstract

**Background:**

In COVID 19 era, the literature on e-learning, or particularly m-learning, has considerably increased focusing on the subject of medical knowledge transfer. Considering the importance of orthopedic knowledge for general practitioners and the inadequacy of the orthopedics internship duration in Mashhad University of Medical Sciences (MUMS), we have developed and investigated a smartphone orthopedic educational application named “Orthobox”.

**Methods:**

In a quasi-clinical before-after trial study, we investigated the benefits of Orthobox application for medical interns attending MUMS orthopedic departments. A total of 120 students (64 and 56 students in control and case groups respectively) were recruited. The application consists of five main parts of medication, common order samples, common prescriptions, cast and splint types, and educational movies. Students who passed the course without getting access to the application (control group) and students who were also using application during the course (case group) were defined, and comparison was done between them objectively through final exam score comparison and subjectively through Visual Analogue Scale (VAS) questionnaire score comparison. Besides, using case group students’ activity report provided by the application panel, correlational analysis was done on their amount of activity on each of the main parts of the application and the corresponding question exam and VAS score separately.

**Results:**

The case group of the study generally achieved higher final exam scores, mainly on *Order* question score (P value<0.001). Total VAS scores were also greater in case group (P value =0.001). It has also been identified that there is a notable positive trend between student’s amount of usage of the application and their final exam scores through correlational analysis. This correlation was not significant about students’ application visit numbers and VAS scores.

**Conclusion:**

These results suggest that m-learning has got the potential to improve students’ medical knowledge and skills by organizing must-to-learn content specified for intern students of orthopedics on one hand, and cause more satisfaction in students about their education on the other hand.

**Trial registration:**

This study was not registered because it is a quasi-clinical trial study.

**Level of evidence:**

Level III (Evidence obtained from well-designed controlled trials without randomization (i.e. quasi-experimental).

**Supplementary Information:**

The online version contains supplementary material available at 10.1186/s12909-021-02918-y.

## Background

Smartphone applications can be the solution to medical students’ struggles for learning high volumes of material in a relatively short time [6, 9]. Recently, the literature on educational e-learning has considerably increased, focusing on the subject of medical knowledge transfer particularly after COVID-19 outbreak [4, 17, 19]. A number of studies have focused on evaluating students’ acceptance and satisfaction of e-learning [1, 8, 10, 15]. Others have shown the benefits of acquiring knowledge or skills by using this method of education [11, 18]. Although some research has been carried out on e-learning [1, 8], there are few if any [14] controlled studies reported on developing, and at the same time investigating the impact of an application (m-learning) on medical students’ knowledge and skills specifically focusing on a single clinical specialty. It thus remains to be studied to what extent m-learning can improve educational quality.

Medical internship courses include mostly practical training at the hospital, but also the self-study of certain related textbooks. Orthopedics is among the most important internship courses for medical students who will frequently encounter patients with orthopedic conditions in clinics and emergency rooms in future (musculoskeletal conditions and injuries account for 137.6 million visits to physicians’ offices and hospital outpatient and emergency departments every year (https://www.perimeterortho.com/what-is-orthopaedics.html)) [2, 5, 7]. Unfortunately, due to the specialized nature of the ward in hospitals and inadequacy of the course duration (2 weeks in Mashhad University of Medical Sciences), students are often confused about practical skills and the necessary knowledge of the field for a general practitioner. In fact, this is one of the most frequently stated problems among them. That is why this paper introduces a new educational method in an attempt to address this problem. In effect, we have developed a smartphone application (app) named “Orthobox” in order to assist interns during their orthopedic course. Decisions about the organization and summarizing the content of this application on orthopedic essentials were made in a way to make it suitable for use not only in student’s free time but also during clinical training and patients’ visit. The main aim of this study is to investigate the influence of this software on orthopedic intern’s knowledge and skills objectively (Exam score) and subjectively (Visual Analogue Scale questionnaire score). As a secondary hypothesis, we were also curious about the correlation between the amount of usage of each part of the software and the related acquired knowledge/skills separately.

## Methods

### Study setting

In a before-after quasi-clinical trial study, conducted in Mashhad University of Medical Sciences (MUMS), orthopedic interns who were participating in the orthopedic course within MUMS educational hospitals were recruited in 2019 over a period of 10 months (divided into two time spans of four months and 2 months of washout period between them). All the experiment protocol of this study was in accordance to Declaration of Helsinki. The study was approved by MUMS Research Ethics Committee (IR.MUMS.REC.1398.065).

### Study design

Using a convenience sampling method, students who entered the orthopedic department for their internship course were recruited. The required sample size was calculated by G*power 3.1.9.4 software. The smallest effect of interest in this study is 5/100. The SD for final exam score was 8/100 which was achieved in a pilot study of six courses before the current study enrollment (effect size of 0.62). Considering type I error of 0.05, test power of 0.90 and ratio allocation of 1, the needed sample size was 55 individuals in each group.

Over the span of the first four months of the research (February 3 to June 5 2019), a total of 65 students were expected to pass the orthopedic course without using the *Orthobox* application who comprise the control group. Each internship course lasts two weeks and includes an average of nine students; during the four months, a total of seven internship courses took place which means a total of around 65 students pass the orthopedic course. Accordingly, fourteen different standard exams were designed by the orthopedic standard exam board before study recruitment began, and seven of them were randomly chosen for our seven groups of 9 students. Then, each group was given one of these seven exams randomly at the end of the two-week course. In a similar manner, after two months of study pause (this will be more clarified in the limitation section), 65 students were expected to participate in the orthopedics course over the span of the second four months of the study (August 5 to December 18 2019) who are considered as the case groups. However, this time, the Orthobox application, available in iOs®- or Android®-based operating systems, was installed on the students’ smartphones. As such, they used the application during the course, and were then tested by the same seven final exam tests given to the control groups. The application panel provided the executors with case group activity reports for further correlational analysis. Both the case and the control group participants were asked to fill out an attitude Visual Analogue Scale (VAS) questionnaire at the end of each course before receiving their scores. Data were also collected from students’ scores on the final exams specifically designed for this research. These data were then gathered and compared with each other in order to identify the impact of the Orthobox on students’ knowledge of orthopedics.

### Tools

#### Orthobox software

**Fig. 1:**
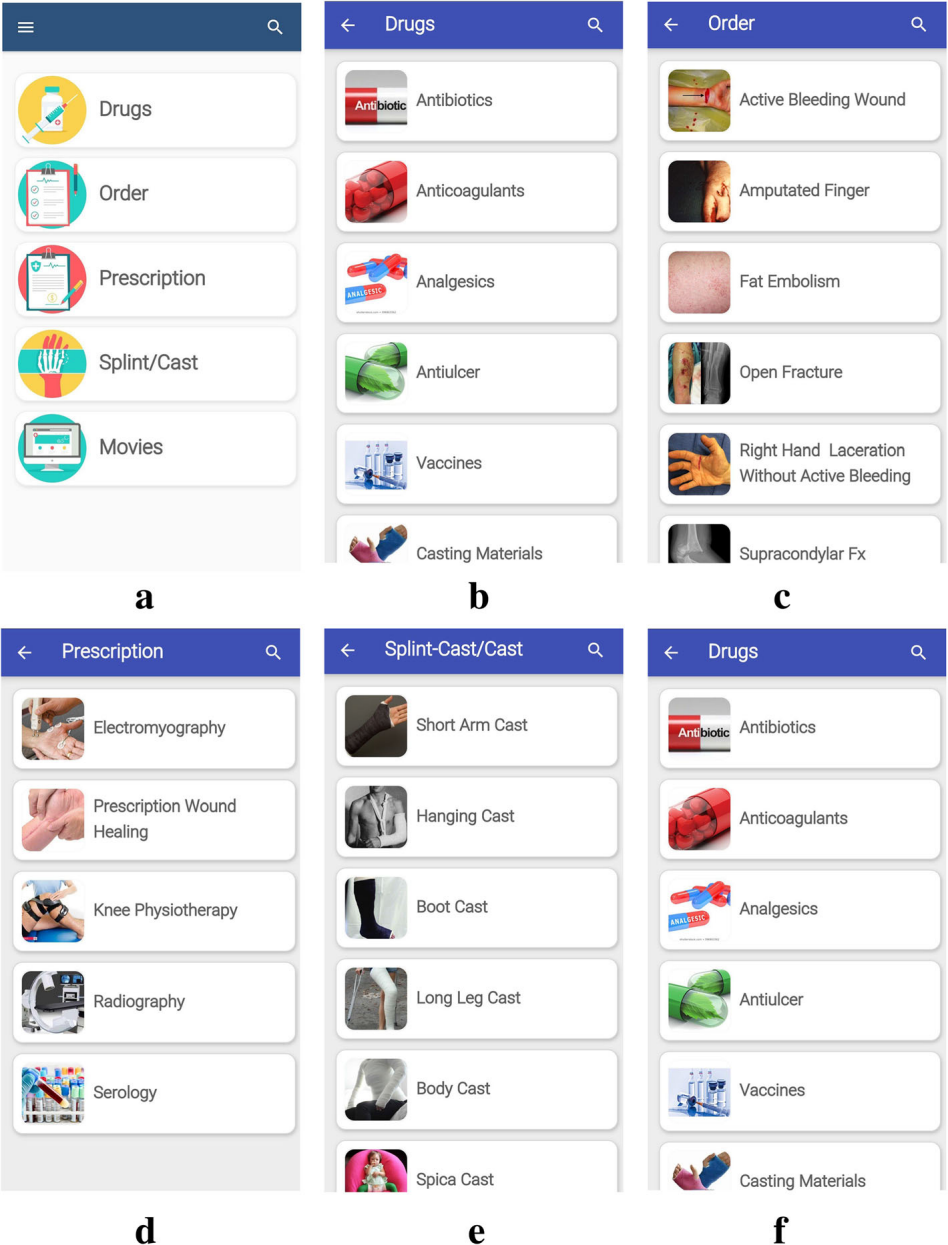
Orthobox Application Pages: a) the main page of the application consists of five parts of *Medicine, Order, Prescription, Cast/Splint* and educational *Movies*; b) the Medicine section consists of different drug categories; c) the Order section includes common orders of the orthopedic hospitalized patients; d) the Prescription section includes common prescriptions of the orthopedic ambulatory patients; e) Cast and splint section consists of the different body parts cast and splints descriptions; f) educational Movies of the common orthopedic procedures

As mentioned before, the interventional tool used for this study was an application specifically designed for teaching orthopedics named *Orthobox*. The *Orthobox* application is **free** of charge. The app consists of five main parts of educational information (Fig. 1A): 1) frequently used medication in orthopedic wards, 2) order samples for common orthopedic hospitalized patients, 3) prescriptions for common orthopedic ambulatory patients, 4) cast and splint types, and 5) educational movies. Here is the detailed description of each part:

1) The first section is mainly a list of several groups of medication including antibiotics, anticoagulants, analgesics, fluid therapy, vaccines, and casting materials. In each group, we have introduced those medicines which are most frequently ordered or prescribed in orthopedic wards or clinics. The available information about each medicine in this application includes name and classification, forms, indications, contraindications, caution, side effects, and group in pregnancy (Fig. 1B). 2) In the second section, we have selected eight common order notes in orthopedic wards, which include orders for the following orthopedic conditions: active bleeding wound, amputated finger, fat embolism, open fracture, laceration without active bleeding, supracondylar fracture, trochanteric fracture, and deep vein thrombosis (DVT). For each order, a typical history of the patient is provided along with their X-ray or real limb image, and also the appropriate order is written at the end. Certain words in the texts of this section are highlighted, which will provide additional information if you click on them (Fig. 1C). 3) The third section presents several frequently used prescriptions for patients with orthopedic conditions such as electromyography, knee physiotherapy, radiology, and serologic tests (Fig. 1D). 4) The section about casts and splints provides specific descriptions along with their related images about different types of casts and splints, which are used in different parts of the body (Fig. 1E). 5) In an attempt to familiarize students with the casting and splinting skills, useful educational movies about upper and lower limb casting and splinting are provided in the last section of the application. This part of the application requires internet connection to work (Fig. 1F). We are going to make the app publicly available once the investigation is complete. An English-language demonstration video of the application is provided as appendix to the paper (Additional file-1). The development of a new platform enables executors to update and customize the content of the app in order to meet the demands of each specific center.

#### Application activity report

Each student of the case group is submitted to the application with a defined username and password which would be expired after two weeks at the end of the course. The Orthobox panel enables the executors to have access to the number of times each user visits each part of the application. At the end of the study, we had an activity report from students in case group so that we could analyze the correlation between student’s exam/ VAS score and his/her activity on application (Additional file-2).

#### Final exam

Both the teaching plan and final exams for orthopedics internship courses are based on 4 divisions: Order, Prescription, Medication and Skill. The final exams are designed by two designated professors from the orthopedics department. The exam material for both the control and case groups was the *Textbook of Orthopedics and Fractures* by Dr B. Aalami Harandi, et al., which students are supposed to self-study. For the purposes of this study, the case groups were also given access to the app as a supplementary source of study. The exam consists of four questions requiring the students to write down a complete order for a certain patient history provided on the exam sheet, write a clinic patient prescription for a presented patient, answer questions regarding a certain medicine, and finally explain the techniques of casting or splinting. Before recruitment, the orthopedics exam board prepared fourteen different, but equally difficult (the level of difficulty was carefully balanced by the in-charge professors), questions for each of the four divisions. These questions were then randomly put together to design 14 written question sheets seven of which were finally selected for students’ final exam in both groups. The exam papers were corrected based on a system of differentiating between major and minor faults. First, the writers carefully defined the standard answer, major fault and minor fault which are reported in Table 1 in detail. This table is based on similar definitions used in driving tests. The term ‘major fault’ is defined here as those answers which miss a necessary point or are completely wrong, whereas the term ‘minor fault’ refers to those answers in which the required point is mentioned but they are not the standard answer. Then, one professor (who was not part of the research team but was involved in the education program routinely) corrected the exam papers based on the determined guideline. Therefore, there was no bias in this regard. This method enabled us to compare the major and minor faults on different exam questions between case and control students, and to analyze their correlation with the number of visits case students had made to each corresponding part of the application.

**Table 1:** Major and minor fault in exam answers

**Definition**		
	**Major**	**Minor**
**Definition**	Answers which miss a necessary point or are completely wrong, leading to a complication for patients if happens in real.	Refers to those answers in which the required point is mentioned but they are not the standard answer.
**Example**		
	**Major**	**Minor**
**Order**	Q: Tibia fracture A: Not ordering for check of the compartment syndrome signs.	Q: Tibia fracture A: Not ordering the ankle and knee joints radiography
**Prescription**	Q: Wound healing discharge A: adult dosing for a child patient.	Q: Wound healing discharge A: Writing “Tab” instead of “Cap” for Cephalexin
**Medication**	Q: Heparin A: Wrong dosing	Q: Heparin A: Not writing complete medicine pharmaceutical forms
**Procedure**	Q: Short leg cast A: Wrong foot angle during casting	Q: Short leg cast A: Wrong but near to correct number of cast layers

In order to determine the reliability of exam questions, we asked the previously designated professor to correct the papers for a second time (test-retest) and also one other staff of the exam board to correct half of the exam papers randomly (interrater). We then analyzed the intra and inter observer reliability between the resultant scores and the prior correction scores. The test-retest and interrater reliabilities were inter-class correlations of > 0.9 and > 0.8, respectively, in all four question types and final score (Table 2).

**Table 2:** Intra and inter observer reliability of the exam scores

Variable	1st rater 1st correction vs 1st rater 2nd correction	1st rater 1st correction vs 2nd rater correction
Order Score	0.963	0.850
Prescription Score	0.958	0.843
Medicine Score	0.953	0.924
Procedure Score	0.977	0.844
Total Score	0.964	0.820

#### Attitude questionnaire

A short VAS scale questionnaire (consisting six question) was designed (Additional file-3). The questions required participants to rate how much they had learned about the different branches of orthopedics knowledge, five questions about medicine, order, prescription, casting and splint skills, so that the amount of learning could be measured quantitatively. Moreover, the last question on this questionnaire asked students about their satisfaction regarding the overall educational programs of the orthopedics course. The ratings given to each question on the questionnaire were then compared between case and control groups to determine their relationship with the application usage. Moreover, the correlation of each VAS question score with number of visits made to the corresponding part in the application within case group students were investigated.

### Study population

Using a convenience sampling method, the orthopedic intern students attending orthopedic departments were recruited for this study. Unfortunately, because of the Covoid-19 pandemic hence changes in educational programs, the last case group was not recruited and we could not reach to the expected 65 students of case group. As a result, a total of 123 students - 66 students in control group and 57 students in case group - participated in this research. Students who did not own a smartphone, who were a guest student, or those who were passing the course for the second time were excluded from the experiment. Two interns in control and one in case group were then excluded according to the criteria of second time participation. Therefore, the final 120 students were studied. Table 3 provides an overview of the study population demographic data. It can be seen from the data that none of the demographic differences were statistically significant between two groups of study. The average score in demographic data refers to the average score of students from the beginning of their studies in medical school. The pre-internship exam is taken twice a year from those students who have passed ten semesters and want to become interns; the score of this exam is also included in the demographic data.

**Table 3:** Demographic data in case and control groups

variable	Whole population	Case group (mean±SD)	Control group (mean±SD)	*P* value
Sex*				
*Women, number (%)*	68 (56.7)	29 (51.8)	39 (60.9)	0.313
*Men, number (%)*	52 (43.3)	27 (48.2)	25 (39.1)	
Age** (year)	25.33	25.30 ± 1.48	25.36 ± 1.26	0.808
Marital status*				
*Married, number (%)*	45 (37.5)	17 (30.4)	28 (43.8)	0.131
*Single, number (%)*	75 (62.5)	39 (69.6)	36 (56.3)	
Internship duration background** (month)	8.07	6.86 ± 5.96	9.28 ± 7.66	0.055
Average score** (0 to 20)	16.05	15.95 ± 1.16	16.15 ± 0.96	0.304
Pre-intenship exam score** (0 to 200)	121.81	119.58 ± 16.63	124.04 ± 23.02	0.252

*mean and SD is calculated using Chi square test for “sex” and “marital status”

** mean and SD is calculated using T test for other quantitative demographic parameters.

### Statistical Analysis

Data management and analysis were performed using the software SPSS 19.0 (2018). Significance levels were set at the 0.05 level using all tests. Case and control groups demographic data are analyzed using T test except for gender and marital status which are based on Chi square test. Students’ quiz scores and VAS scores are analyzed using T test in order to compare the scores between two groups. Pearson’s and Spearman Correlations was used to analyze the association of factors (application visit numbers, final exam score and VAS questionnaire score). We analyzed the intra observer reliability of the exam scores (two correction scores by one rater) and inter observer reliability (two correction scores by two different raters) using ICC tests.

## Results

The first set of analyses determines the effectiveness of the application on intern’s knowledge. Table 4 compares the final score, minor and major error of each question of the exam separately between two groups. What stands out in the table is that the case group earned significantly higher final exam scores and lower final minor and major errors compared to the control group (P value <0.001). From this data, we can see that *Order* section of the application resulted in the most effect on the corresponding indicators (P value <0.001). The effect on *Procedure* and *Medicine* knowledge are significant in minor error indicators only (P value <0.001 and =0.001 respectively) but there is no noticeable difference in the other two indicators of these sections between two groups (Table 4).

**Table 4:** Quiz score comparison between Case and Control groups

Variable	Case group (mean±SD)	Control group (mean±SD)	P value
**Medicine knowledge**			
*Minor pitfall*	0.73 ± 0.84	1.27 ± 0.89	**0.001**
*Major pitfall*	1.19 ± 1.11	1.06 ± 1.10	0.500
*Final score*	20.67 ± 3.44	20.53 ± 3.16	0.810
**Order knowledge**			
*Minor pitfall*	1.80 ± 1.46	3.56 ± 1.60	**<0.001**
*Major pitfall*	1.80 ± 1.72	3.40 ± 1.76	**<0.001**
*Final score*	17.78 ± 5.73	11.23 ± 5.37	**<0.001**
**Prescription knowledge**			
*Minor pitfall*	0.51 ± 0.73	0.63 ± 0.67	0.380
*Major pitfall*	0.75 ± 0.57	0.84 ± 0.68	0.410
*Final score*	22.23 ± 1.89	21.83 ± 2.00	0.260
**Procedure knowledge**			
*Minor pitfall*	0.23 ± 0.46	1.07 ± 0.87	**<0.001**
*Major pitfall*	0.94 ± 0.92	1.03 ± 0.91	0.610
*Final score*	21.92 ± 2.57	20.83 ± 2.64	0.230
**Final score**			
*Minor pitfall*	3.28 ± 2.06	6.55 ± 2.41	**<0.001**
*Major pitfall*	4.69 ± 2.49	6.33 ± 2.54	**0.001**
*Final score*	82.62 ± 7.97	74.43 ± 8.16	**<0.001**

Turning now to the impact of application on student’s learning self-assessment and satisfaction measured by VAS questionnaire, the mean total scores have a significant difference (P value=0.001). The results, as demonstrated in Table 5, indicate that the *order* and *procedure* VAS scores also differ notably (P value<0.001 and =0.008 respectively) (Table 5).

**Table 5:** VAS questionnaire comparison between case and control groups

Variable	Case (mean±SD)	Control (mean±SD)	*P* value
***VAS-Medicine knowledge (0 to 10)***	7.45 ± 1.54	6.89 ± 1.95	0.09
***VAS-Order (0 to 10)***	7.83 ± 1.40	6.55 ± 2.02	**<0.001**
***VAS-Prescription (0 to 10)***	7.54 ± 1.76	6.94 ± 1.76	0.067
***VAS-Splint and casting (0 to 10)***	6.38 ± 2.37	5.11 ± 2.83	**0.008**
***VAS-Satisfaction (0 to 10)***	7.59 ± 2.00	6.87 ± 2.36	0.089
***VAS-Total (0 to 10)***	7.18 ± 1.56	6.03 ± 2.02	**0.001**

The next section of the survey was concerned with the case group. The results of the correlational analysis between each case student’s number visits to different app sections and the corresponding exam and VAS score is shown in Tables 6 and 7. The visit numbers to *Medicine* and *Prescription* sections of the app positively correlates with the corresponding final scores (P value= 0.028 and P value= 0.01 respectively). Furthermore, the *Prescription* major error negatively correlates with the visit numbers (P value= 0.019) (Table 6). No significant correlation is found between VAS score indicators and the number of corresponding app section visits (Table 7).

**Table 6:** Correlation of the application visit number with corresponding exam scores

Variable	r coefficient	*P* value
**Medicine knowledge**	**Medicine Visit Number**	
*Minor error*	-0.248	0.065
*Major error*	-0.239	0.076
*Final score*	0.293	**0.028**
**Order knowledge**	**Order Visit Number**	
*Minor error*	0.083	0.544
*Major error*	-0.106	0.438
*Final score*	0.074	0.589
**Prescription knowledge**	**Prescription Visit Number**	
*Minor error*	-0.144	0.289
*Major error*	-0.313	**0.019**
*Final score*	0.343	**0.01**
**Procedure knowledge**	**Procedure Visit Number**	
*Minor error*	0.111	0.415
*Major error*	-0.104	0.444
*Final score*	0.105	0.441

**Table 7:** Correlation of the application visit number with corresponding VAS scores

Variable	r coefficient	P value
	**Medicine Visit Number**	
**Medicine VAS**	0.056	0.68
	**Order Visit Number**	
**Order VAS**	-0.104	0,445
	**Prescription Visit Number**	
**Prescription VAS**	-0.168	0.217
	**Procedure Visit Number**	
**Procedure VAS**	0.141	0.3

*Medicine* section was the most visited part of which Antibiotics (Penicillin G) contributes the most portion. Of *Orders* and *Prescriptions*, “Active Bleeding Wound” and “Wound Healing Prescription” were the most seen pages respectively (Additional file-2, Table 8).

**Table 8:** Frequency of each part of the application visit number

App Section	Visit Number	Subsection	Visit Number
**Medicine**	2014		
		Analgesic	253
		Antibiotic	827
		Antiulcer	55
		Anticoagulant	239
		Serum	239
		Vaccine	136
		Casting Material	265
**Movie**	212		
**Order**	1336		
**Prescription**	777		
**Cast/Splint**	1530		

## Discussion

Prior studies have noted the importance of e-learning and smartphone applications for medical education [6, 9]. However, no data was found on the association between an application amount of usage and student’s knowledge in different parts of an app separately in detail similar to the present study.

The first study question was about the application impact on student’s clinical knowledge. The most obvious finding to emerge from the analysis is a remarkable difference in students’ final exam scores between two groups. The *Order* section of the app had the greatest impact on its exam indicators. Hence, it could conceivably be hypothesized that medical students need more education and training on order writing basics and tips which can be done by means of m-learning. *Procedure* and *Medicine* parts of the app mainly caused decrease in minor errors which probably means that reviewing the application content - being always available - by watching movies and scanning the prescriptions helped with memorization of the details of the casting/splinting skills and prescription medicine and dosing. It is encouraging to compare this figure with that found by Martinez et al. (2017) who held a randomized trial among eighty interns to evaluate an application designed to review key concepts in Internal Medicine; the app contents were in a format similar to national examination for practicing medicine in Chile; they found that clinical performance was significantly improved among medical interns based on the mean change in overall score and the average time required to answer [13]. This finding is also consistent with that of Bonabi et al.’s (2019) randomized trial which investigated a newly designed evidence-based smartphone application for 107 public health service physicians (PHS physicians) who were assigned randomly to intervention and control groups; they reported an improvement in knowledge, attitude and practice in post-intervention data, but the difference between two groups were not significant. In this study, a subjective self-administered questionnaire was used as the measurement tool which probably was not strong enough to assess the physicians’ knowledge and practice [3].

On the question of application effect on interns’ self-assessment of their learning and satisfaction (VAS questionnaire score), this study found that the intervention students assessed their learning notably better and were more satisfied with their education than the control students. It can thus be suggested that summarizing the essential information in the form of application for medical interns guides them toward learning the most needed skills, organizes their minds and, therefore, helps them better manage common patients, which consequently makes them more satisfied with their understandings. In accordance with the present results, Zhang et al. (2016) have demonstrated the advantages of a smartphone application in higher levels of education for delirium inter-professional education. After 4 weeks of implementation of the application, the participants took part in focus group discussions and stated that the application was appealing to them; two focus groups were held with 3 and 7 participants in each group; it was proposed that this is due to the ability of the software to provide structured material in a highly accessible manner [20]. Looking at a different aspect, Lameris et al. (2015) performed an investigation on the impact of a formative testing application on 461 medical students’ study behavior. The app invited students to complete 7 formative tests throughout their course; the study was based on the difference of students’ study time and grades in groups of intensive users, moderate users and non-users; they finally found it an attractive way of intervention to stimulate study behavior [12].

The third question in this research is whether the amount of visiting each part of the app correlates with the corresponding exam question score. It is interesting to note that *Medicine* and *Prescription* final exam scores show significant correlation. In *Orde*r and *Procedure* parts, there is a negative correlation between number of visits and number of major errors, but a positive correlation with number of minor errors. These results probably mean that using the app caused a partial conversion of major to minor errors on students’ answer sheets; however, the correlations were not significant. With respect to the last research question about the association between the amount of visiting each part of the app and the corresponding student’s self-assessment VAS score within case group, it was found that there is no association between these two parameters in spite of the significant difference between case and control groups comparison of VAS scores. Therefore, since mere access to the app increased students’ VAS scores, it can be argued that that quantity of the application usage was not effective on learning self–assessment.

The least visited part of the application by the case group was *Movies*. Considering the Orthobox application is intended for learning on-the-go and during practice, a possible explanation for this result might be that *Movies* was the only part of the app which required internet connection. It can therefore be assumed that fully offline medical applications would be more accepted and practical particularly during clinical practice where internet connection may not be available. This result supports previous research of Sujan et al. (2010) indicating how poor information infrastructure and higher costs of internet connectivity in developing countries points out the importance of developing mobile offline learning [16].

### Study limitation

Unfortunately, case group enrollment coincided with the COVID-19 virus pandemic hence changes in educational programs, so we could not recruit the last group of case students. Although the designed final exam questions were based on the routine orthopedic four-question exam structures, the availability of application as an extra source of study for case group students - while control group did not get access to the app - may cause bias. However, we did our best to equalize the situation for two groups of the study by giving them the same seven designed question sheets which were based on routine orthopedic final exam structure. By considering two months of study pause, we tried to reduce the probability of questions to be exposed for the case group, considering the routine behavior of students looking for previous course question samples to study. One application limitation is that it was only able to report each user visits to different pages of the app, and, unfortunately, it was not possible to detect the length of time the user spent on each visit. To control factors that threaten the internal validity of a quasi-experimental study, we gathered demographic data on students’ previous performances based on their average and pre-internship scores, and noted no notable difference between two groups of study (Table 3). Since both groups of case and control were not passing the orthopedic course at the same time, they were blinded to the identity of the students in the other group. None of students discontinued the study. With regard to reactivity, since all students in case and control groups were aware of their participation, this factor affects all of the students equally.

## Conclusion

The main goal of the current study was to determine the utility of the designed orthopedic application (Orthobox) for medical interns and its impact on their knowledge and skills. This study has identified that the students who used the application got higher scores, specifically, with regard to *Order* question. The effect on other questions were mainly on reducing minor faults. A significant positive trend between student’s application usage and their final exam and VAS scores was also noted. This study is the first comprehensive investigation into an educational application in MUMS and may be of interest to other researchers designing similar applications.

To develop a full picture of smartphone application effect on medical education, additional studies will be needed to determine its utility in other medical courses and also for higher levels of education (specialty or fellowship).

### Ethical Consideration

Informed written consent was obtained from all students (Additional files 5 and 6) in which students confirm that their participation in this research is entirely voluntary and that confidential information will not be shared. One code was defined for each student during data analysis for the reason of information privacy. The department educational protocols were completely addressed during the study. It also cannot be considered unethical that case group students acquired higher scores. This is mostly because higher scores are not important in their future exams or achievements, and the two groups were not competing with each other.

## Supplementary Information

**Additional file 1.** The application demonstration video. Given the issue of language difference, we made a short English-language demonstration video.

**Additional file 2.** The times of Orthobox app usage by case group students is provided in an excel datasheet.

**Additional file 3. **Attitude VAS scale questionnaire.

**Additional file 4.** Ethics Committee Approval.

**Additional file 5.** Informed Consent Form (Farsi).

**Additional file 6.** Informed Consent Form (English).

## Data Availability

Correspondence and requests for materials should be addressed to Moradial@mums.ac.ir.

## Abbreviations

app: Application
MUMS: Mashhad University of Medical Sciences
VAS: Visual Analogue Scale
SPSS: Statistical Package for the Social Sciences
