# Supplementary figures and images for: Investigating a Newly Developed Educational Orthopedic Application for Medical Interns in a Before-after Quasi-clinical Trial Study
Source: BMC Med Educ. 2021 Sep 29;21:515. doi: 10.1186/s12909-021-02918-y (PMC8480122; doi:10.1186/s12909-021-02918-y)

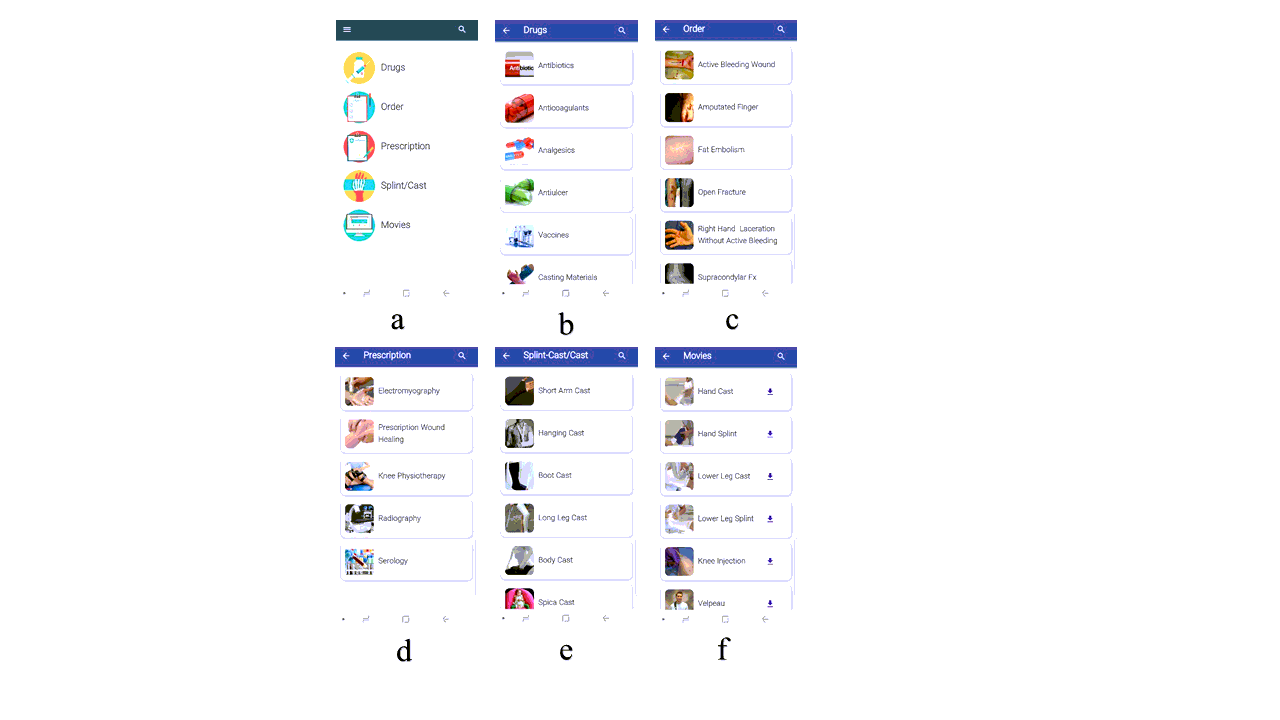

Supplement: Supplementary file 2 — Additional file 2. The times of Orthobox app usage by case group students is provided in an excel datasheet. [file 12909_2021_2918_MOESM2_ESM.png]
