# Supplementary material for: Investigating a Newly Developed Educational Orthopedic Application for Medical Interns in a Before-after Quasi-clinical Trial Study
Source: BMC Med Educ. 2021 Sep 29;21:515. doi: 10.1186/s12909-021-02918-y (PMC8480122; doi:10.1186/s12909-021-02918-y)
Supplement: Supplementary file 3 — Additional file 3. Attitude VAS scale questionnaire. [file 12909_2021_2918_MOESM3_ESM.pdf]

Orthopedic Interns Final Learning Self-assessment

First Name: **Family name:** **Age:** **Marital status:**

Internship duration (month): **Average score:** **Pre-internship exam score:**

*Please, answer to the questions based on the visual scale below. Zero represents not learning any skills and ten learning required skills completely. For example, if you assess your learning scale as high, answer like this:

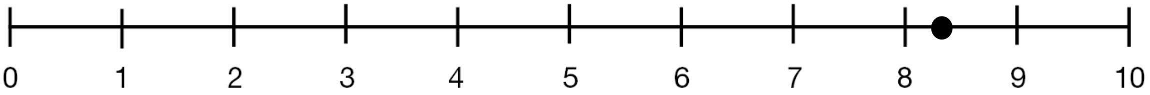

not at all **Very much**

1) How much have you get learned about the frequently prescribed or ordered medicines in orthopedic department?

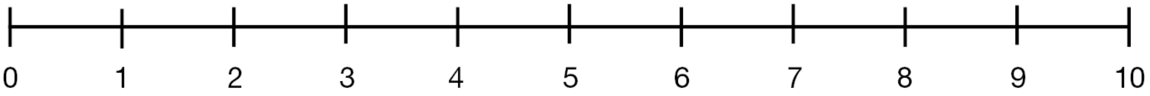

not at all **Very much**

2) How much you get mastery of writing urgent patients' orders- like open tibia fracture, intertrochanteric fracture and finger amputation-at the end of orthopedic course?

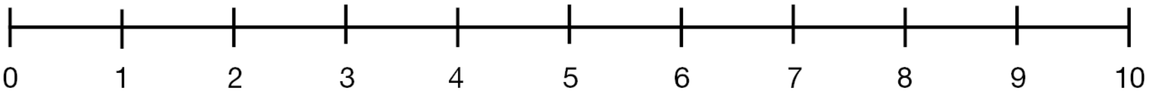

not at all **Very much**

3) Are you enable to write prescription for orthopedic frequently visited ambulatory patients?

0 1 2 3 4 5 6 7 8 9 10

not at all **Very much**

4) How much are you skilled in lower limb splinting and casting at the end of the orthopedic course?

0 1 2 3 4 5 6 7 8 9 10

not at all **Very much**

5) How much are you skilled in upper limb splinting and casting at the end of the orthopedic course?

0 1 2 3 4 5 6 7 8 9 10

not at all **Very much**

6) How much satisfied are you with the orthopedic course education programs in general?

0 1 2 3 4 5 6 7 8 9 10

not at all **Very much**

Thanks for your cooperation!
