# Supplementary material for: Investigating a Newly Developed Educational Orthopedic Application for Medical Interns in a Before-after Quasi-clinical Trial Study
Source: BMC Med Educ. 2021 Sep 29;21:515. doi: 10.1186/s12909-021-02918-y (PMC8480122; doi:10.1186/s12909-021-02918-y)
Supplement: Supplementary file 4 — Additional file 4. Ethics Committee Approval. [file 12909_2021_2918_MOESM4_ESM.pdf]

Mashhad University of Medical Sciences

Research Ethics Certificate

Approval ID:	IR.MUMS.REC.1398.065	Approval Date:	2019-05-25
Evaluated by:	Mashhad University of Medical Sciences		
Status:	Approved		
Approval Statement:	The project was found to be in accordance to the ethical principles and the national norms and standards for conducting Medical Research in Iran.<br>Notice:<br>1. Although the proposal has been approved by the research ethics committee, meeting the professional and legal requirements is the sole responsibility of the PI and other project collaborators.<br>2. This certificate is reliant on the proposal/documents received by this committee on 2019-05-25. The committee must be notified by the PI as soon as the proposal/documents are modified.		
Proposal Title:	Investigating a Newly Developed Educational Orthopedic Application for Medical Interns in Mashhad University of Medical Sciences		
Principal Investigator:	Name: Ali Moradi<br>Email: moradial@mums.ac.ir		

Dr. Mohammadreza Darabi mahboub
Director of University/Regional Research Ethics Committee
Mashhad University of Medical Sciences

Dr. Mohsen Tafaghodi
Secretary of University/Regional Research Ethics Committee
Mashhad University of Medical Sciences
