# Supplementary material for: Investigating a Newly Developed Educational Orthopedic Application for Medical Interns in a Before-after Quasi-clinical Trial Study
Source: BMC Med Educ. 2021 Sep 29;21:515. doi: 10.1186/s12909-021-02918-y (PMC8480122; doi:10.1186/s12909-021-02918-y)
Supplement: Supplementary file 5 — Additional file 5. Informed Consent Form (Farsi). [file 12909_2021_2918_MOESM5_ESM.docx]

**رضايت نامه شرکت در طرح بررسی تاثیر استفاده از نرم افزارکمک آموزشی مبتنی بر تلفن همراه برمیزان یادگیری کارورزان پزشکی عمومی در یک دوره یک و نیم ساله در مشهد**

**آقاي/ خانم محترم**

بدين وسيله از شما جهت شركت در پژوهش فوق‌الذکر دعوت به عمل مي‌آيد. اطلاعات مربوط به این پژوهش در این برگه خدمتتان ارائه شده است و شما برای شرکت یا عدم شرکت در این پژوهش آزاد هستید.

شما مجبور به تصميم گيري فوري نيستيد و برای تصميم گيري در اين باره مي‌توانيد سوالات خود را از تيم پژوهشي بپرسيد و با هر فردي که مایل باشید مشورت نماييد. قبل از امضاي اين رضايت نامه مطمئن شويد كه متوجه تمامي اطلاعات اين فرم شده‌ايد و به تمام سوالات شما پاسخ داده شده است.

**مجري پژوهش**

1. من مي‌دانم كه اهداف اين پژوهش عبارتند از:

طراحی نرم افزارکمک آموزشی ارتوباکس مبتنی بر تلفن همراه در خصوص بیماریهای شایع ارتوپدی قابل استفاده برای کارورزان

تولید نرم افزارکمک آموزشی ارتوباکس مبتنی بر تلفن همراه در خصوص بیماریهای شایع ارتوپدی قابل استفاده برای کارورزان

تعیین اثر بکار گیری نرم افزارکمک آموزشی ارتوباکس در وضعیت آموزشی دانش داروشناسی در حیطه ارتوپدی کارورزان رشته پزشکی عمومی در بخش ارتوپدی

تعیین اثر بکار گیری نرم افزارکمک آموزشی ارتوباکس در وضعیت آموزشی دانش اوردر نویسی در حیطه ارتوپدی کارورزان رشته پزشکی عمومی در بخش ارتوپدی

تعیین اثر بکار گیری نرم افزارکمک آموزشی ارتوباکس در وضعیت آموزشی دانش نسخه نویسی در حیطه ارتوپدی کارورزان رشته پزشکی عمومی در بخش ارتوپدی

تعیین اثر بکار گیری نرم افزارکمک آموزشی ارتوباکس در وضعیت آموزشی دانش مهارتهای بالینی در حیطه ارتوپدی کارورزان رشته پزشکی عمومی در بخش ارتوپدی

1. من مي­دانم که شرکت من در اين پژوهش کاملاً داوطلبانه است و مجبور به شرکت در اين پژوهش نيستم.

به من اطمينان داده شد که اگر حاضر به شركت در اين پژوهش نباشم، از مراقبت‌هاي معمول تشخيصي و درماني محروم نخواهم شد و رابطه درماني من با مركز درماني و پزشك معالجم دچار اشكال نمي‌شود.

1. من مي‌دانم كه حتي پس از موافقت با شركت در پژوهش مي‌توانم هر وقت كه بخواهم، پس از اطلاع به مجري، از پژوهش خارج شوم و خروج من از پژوهش باعث محرومیت از دریافت خدمات درمانی معمول برای من نخواهد شد.
2. نحوه‌ي همکاري اينجانب در اين پژوهش به اين‌صورت است:

ابزار مداخله ای استفاده شده در این مطالعه نرم افزار طراحی شده جهت آموزش مجازی ارتوپدی میباشد. کارورزان در شش ماهه دوم، در ابتدای دوره نرم افزار آموزشی که در دو ورژن اندرویید و IOS طراحی شده است بر روی تلفن های همراه کارورزان نصب میشود. کارورزان با استفاده از نرم افزار اموزشی دوره ارتوپدی را میگذرانند و گروه مورد مطالعه را تشکیل میدهند.

جهت **اعتبارسنجی و ارزشیابی** نرم افزار در انتهای هر دوره میزان فراگیری دانشجویان از مباحث مختلفی که در نرم افزار گنجانده شده با استفاده از پرسشنامه ای که به صورت معیار VAS (visual analog scale) طراحی شده بود از ایشان پرسیده میشود.

جهت ارزیابی مفید بودن نرم افزار ما از ابزار دیگری که امتحان پایان بخش کارورزان به صورت کتبی است نیز استفاده میکنیم و ۱۴ سری امتحان استاندارد تعریف کرده و به صورت تصادفی از گروه شاهد و مورد گرفته میشود و اشتباهات کارورزان به صورت اشتباهات اصلی و جزیی به روش کور ثبت میشود.

1. منافع احتمالي شرکت اينجانب در اين مطالعه به اين شرح است:

استفاده از یک نرم افزار کمک آموزشی ارتوپدی در طول دوره و یادگیری بهتر مفاهیم مهم و کاربردی .

1. آسيب‌ها و عوارض احتمالي شرکت در اين مطالعه به اين شرح است:

ندارد

1. در صورت عدم تمایل به شرکت در مطالعه روش معمول درمانی برای من ارائه خواهد شد که منافع و عوارض آن به این شرح است: مصداق ندارد.
2. من مي­دانم كه دست اندر كاران اين پژوهش، كليه اطلاعات مربوط به من را نزد خود به صورت محرمانه نگه‌داشته و فقط اجازه دارند فقط نتايج كلي و گروهي اين پژوهش را بدون ذکر نام و مشخصات اينجانب منتشر كنند.
3. می­دانم که كميته اخلاق در پژوهش با هدف نظارت بر رعایت حقوق اينجانب مي‌تواند به اطلاعات من دسترسي داشته باشد.
4. من مي‌دانم كه هيچ‌يک از هزينه‌هاي انجام مداخلات پژوهشي به شرح ذيل بر عهده من نخواهد بود.

نصب نرم افزار بر روی تلفن همراه

1. خانم / آقاي ..مهلا دلیری...................جهت پاسخگويي به اينجانب معرفي شد و به من گفته شد تا هر وقت مشكلي يا سوالي در رابطه با شركت در پژوهش مذكور پيش آمد با ايشان در ميان بگذارم و راهنمايي بخواهم.

آدرس و شماره تلفن ثابت و همراه ايشان به شرح به من ارائه شد:

- **آدرس:** ....مشهد- دانشکده ی علوم پزشکی مشهد- بیمارستان قائم- مرکز تحقیقات ارتوپدی **...................................................................................................................................................**
- **تلفن ثابت: ....05136110352......................................................................**
- **تلفن همراه: ........09351079507..................................................................**

1. من مي‌دانم كه اگر در حين و بعد از انجام پژوهش هر مشكلي اعم از جسمي و روحي به علت شرکت در اين پژوهش براي من پيش آمد درمان عوارض، و هزينه‌هاي آن و غرامت مربوطه بر عهده مجري خواهد بود.
2. من مي­دانم اگر اشکال يا اعتراضي نسبت به دست اندركاران يا روند پژوهش دارم مي­توانم با كميته اخلاق در پژوهش دانشگاه علوم پزشکی مشهد به آدرس: **خیابان دانشگاه- جنب سینما هویزه- ساختمان قرشی- معاونت پژوهش و فناوری دانشگاه** تماس گرفته و مشکل خود را به صورت شفاهي يا كتبي مطرح نمايم.
3. اين فرم اطلاعات و رضايت آگاهانه در دو نسخه تنظيم شده و پس از امضا يک نسخه در اختيار من و نسخه ديگر در اختيار مجري قرار خواهد گرفت.

اينجانب ........................... موارد فوق‌الذکر را خواندم و فهميدم و بر اساس آن رضايت آگاهانه خود را براي شركت در اين پژوهش اعلام مي‌کنم.

امضاي و اثر انگشت شركت كننده

اينجانب ........................... ولی/قیم قانونی ............................ موارد فوق‌الذکر را خواندم و فهميدم و بر اساس آن رضايت آگاهانه خود را براي شركت کودک/ فرد تحت سرپرستی من در اين پژوهش اعلام مي‌کنم.

امضاي و اثر انگشت ولی/ قیم قانونی

اينجانب …مهلا دلیری…………… خود را ملزم به اجراي تعهدات مربوط به مجري در مفاد فوق دانسته و متعهد مي‌گردم در تأمين حقوق و ايمني شركت كننده در اين پژوهش تلاش نمايم.

مهر و امضاي مجري پژوهش
