# Supplementary material for: Investigating a Newly Developed Educational Orthopedic Application for Medical Interns in a Before-after Quasi-clinical Trial Study
Source: BMC Med Educ. 2021 Sep 29;21:515. doi: 10.1186/s12909-021-02918-y (PMC8480122; doi:10.1186/s12909-021-02918-y)
Supplement: Supplementary file 6 — Additional file 6. Informed Consent Form (English). [file 12909_2021_2918_MOESM6_ESM.docx]

**Informed Consent form for Investigating a Newly Developed Educational Orthopedic Application for Medical Interns**

This Informed Consent Form is for students who attend orthopedic department for their orthopedic course.

I am going to give you information and invite you to be part of this research. You do not have to decide today whether or not you will participate in the research. Before you decide, you can talk to anyone you feel comfortable with about the research.

The study executor

1. **I know the purposes of this study are as follows:**

Educational Orthobox application design for medical interns

Educational Orthobox application development for medical interns

Investigating the effect of the application on students’ orthopedic pharmaceutical knowledge

Investigating the effect of the application on students’ knowledge about common orthopedics hospitalized patients’ order writing.

Investigating the effect of the application on students’ knowledge about common orthopedics ambulatory patients’ prescription writing.

Investigating the effect of the application on students’ clinical skills knowledge

1. **I know** that my participation in this research is entirely voluntary. It is my choice whether to participate or not.

I know whether I choose to participate or not, all the services I receive at this clinic will continue and nothing will change.

1. **I know** if I choose not to participate in this research project, I will be offered the treatment that is routinely offered in this clinic/hospital, and we will tell you more about it later. You may change your mind later and stop participating even if you agreed earlier.
2. **Intervention Description:**

During my orthopedic course, the Orthobox application will be installed on my phone and at the end of the course I will took the final exam and fill out a VAS questionnaire sheet.

1. **Benefits**

A free educational application will be installed on my phone to learn better the orthopedic high yield content for a general practitioner.

1. **Risks**

The study does not include any risks (e.g. data privacy warning)

1. **I know** if I decide not to take part in this research, all of my rights will still be respected and my treatment at this clinic will not be affected in any way, the risks and benefits of which are as follows:

**Not applicable**

1. **I know** that confidential information will not be shared. There will be small meetings in the community and these will be announced. After these meetings, the results will be published in order that other interested people may learn from our research.
2. **I know** that the Ethics Research Committee can get access to my data with the purpose of supervising and protecting my rights
3. **I know** that I am not responsible for any of the study intervention costs which are as follows:

Application installation on phone

1. **I know** that ***Ms. Mahla Daliri*** is introduced to me to ask questions about the study, and all the questions asked by me have been answered correctly and to the best of her ability. I confirm that the individual has not been coerced into giving consent, and the consent has been given freely and voluntarily:

**Address**: Orthopedic Research Center, Ghaem hospital, Mashhad University of Medical Sciences, Mashhad, Iran.

**Telephone:** 05136110352

**Mobile:** 09351079507

1. **I know** that if due to study participation, something unexpected including physical or psychologic complications happen, the study team will provide me with all treatment cost and compensation.
2. **I know** that all my objections to executors or study process will be addressed by Mashhad Ethics Research Committee.
3. A copy of this ICF has been provided to me.

Me….[Participant Name] have read the foregoing information, or it has been read to me. I have had the opportunity to ask questions about it and any questions that I have asked have been answered to my satisfaction. I consent voluntarily to participate as a participant in this research.

**Signature of Participant ___________________**

**Date ___________________________**

**Day/month/year**

Me ***Mahla Daliri*** have accurately read out the information sheet to the potential participant, and to the best of my ability made sure that the participant understands that the following will be done:

**Signature of Researcher /person taking the consent__________________________**

**Date ___________________________**

**Day/month/year**
